# The selectivity of rostroventral medulla descending control of spinal sensory inputs shifts postnatally from A fibre to C fibre evoked activity

**DOI:** 10.1113/jphysiol.2013.267518

**Published:** 2014-02-17

**Authors:** Stephanie C Koch, Maria Fitzgerald

**Affiliations:** Department of Neuroscience, Physiology and Pharmacology, UCLLondon, WC1E 6BT, UK

## Abstract

Brainstem descending control is crucial in maintaining the balance of excitation and inhibition in spinal sensory networks. In the adult, descending inhibition of spinal dorsal horn circuits arising from the brainstem rostroventral medial medulla (RVM) is targeted to neurons with a strong nociceptive C fibre input. Before the fourth postnatal week, the RVM exerts a net facilitation of spinal networks but it is not known if this is targeted to specific dorsal horn neuronal inputs. As the maturation from descending facilitation to inhibition occurs only after C fibre central synaptic maturation is complete, we hypothesized that RVM facilitation in young animals is targeted to A fibre afferent inputs. To test this, the RVM was stimulated while recording dorsal horn neuronal activity *in vivo* under isoflurane anaesthesia at postnatal day (P) 21 and P40 (adult). Electrical thresholds for A and C fibre evoked activity, spike counts and wind-up characteristics at baseline and during RVM stimulation (10–100 µA, 10 Hz) were compared. In adults, RVM stimulation selectively increased the threshold for C fibre evoked activity while at P21, it selectively decreased the threshold for A fibre evoked activity and these effects were correlated to the wind-up characteristics of the neuron. Thus, the postnatal shift in RVM control of dorsal horn circuits is not only directional but also modality specific, from facilitation of A fibre input in the young animal to inhibition of nociceptive C input in the adult, with additional contextual factors. The descending control of spinal sensory networks serves very different functions in young and adult animals.

## Introduction

The importance of supraspinal modulation of spinal nociceptive networks has been widely reported and shown to arise from a number of brain regions (Basbaum & Fields, 1984; Fields *et al*. 2006), but research in the area has largely focused on projections from the midbrain periaqueductal grey and the brainstem region of the rostroventral medial medulla (RVM). The RVM acts as a common output from which supraspinal sites can exert modulatory control over spinal nociceptive circuits (see Fields *et al*. 2006) and, along with noradrenergic pontine nuclei, the site through which the periaqueductal grey exerts its spinal effects (Gebhart *et al*. 1983; Prieto *et al*. 1983; Odeh & Antal, 2001). The ventral medulla and the periaqueductal grey also receive direct afferent input from neurons in the superficial dorsal horn (Todd *et al*. 2000) suggesting a spinobulbospinal loop that regulates spinal nociceptive activity (Tavares & Lima, 1994). Importantly, descending control from the brainstem is biphasic and dependent upon stimulus strength and ongoing supraspinal activity; although early studies mainly reported the descending inhibitory actions of the brainstem (Fields *et al*. 1977; Sandkuhler & Gebhart, 1984), it is now widely accepted that projections from the same areas can also result in facilitation of nociceptive reflexes depending on stimulus strength or concentration of drug used (Zhuo & Gebhart, 1997). Descending facilitation can be evoked by RVM stimulation at low stimulus intensity (e.g. 2–20 µA) in the adult rat and has been shown to be a major contributor in neuropathic and inflammatory pain states, where the equilibrium between excitation and inhibition appears to be disrupted (Herrero *et al*. 2000; Porreca *et al*. 2002).

Both anterograde and retrograde labelling in the rat show that descending projections from the RVM terminate within laminae I, II and V where neurons also receive powerful mono-and polysynaptic inputs from Aδ and C fibre nociceptive primary afferents (Todd, 2010; Aicher *et al*. 2012); interestingly however, inhibition arising from the RVM is selective, being preferential to C fibre input (Lu *et al*. 2004).

Descending inhibitory controls are known to be immature at birth (Fitzgerald & Koltzenburg, 1986; Hathway *et al*. 2009; Kwok *et al*. 2013) but little is known of the functional connectivity of brainstem dorsal horn circuits over early postnatal development. Early studies reported that stimulation of the dorsolateral funiculus in young animals caused little or no supraspinal inhibition of spinal dorsal horn neuronal activity before postnatal day (P)10, with a slow maturation over the following weeks (Fitzgerald & Koltzenburg, 1986). More recent studies, focusing upon nociceptive reflexes, have shown that graded electrical stimulation of the RVM does not inhibit nociceptive reflex electromyographic activity until the fourth postnatal week, the influence of the RVM over reflexes being primarily facilitatory rather than inhibitory before that age (Hathway *et al*. 2009).

In adult rats, descending inhibition arising from the brainstem selectively dampens the activity of wide dynamic range dorsal horn neurons with strong C fibre input (McMullan & Lumb, 2006; Waters & Lumb, 2008). Interestingly, C fibre afferent input to the dorsal horn is weak at birth and slowly strengthens over the following 3–4 weeks, as the exuberant immature A fibre input is refined (Beggs *et al*. 2002; Granmo *et al*. 2008), over the same timeline as the development of descending inhibition (Fitzgerald & Koltzenburg, 1986). Maturation of descending inhibition appears to require normal C fibre input; ablation of afferent C fibres by neonatal capsaicin treatment prevents the maturation of descending inhibitory control later in life (Cervero & Plenderleith, 1985; Zhuo & Gebhart, 1994). We hypothesize that the dorsal horn neuronal selectivity of descending brainstem pathways differs in young and adult animals. The predominant A fibre dorsal horn input in the first postnatal weeks (Park *et al*. 1999; Nakatsuka *et al*. 2000) and the propensity towards descending facilitation before P28 (Hathway *et al*. 2009; Kwok *et al*. 2013), suggests that RVM stimulation may be focused upon enhancing A fibre evoked activity rather than inhibiting C fibre input in the immature dorsal horn.

To test this hypothesis we have investigated the functional effects of direct electrical stimulation of the RVM upon dorsal horn cell activity in anaesthetized young and adult rats *in vivo*. The selectivity of RVM control upon A fibre and C fibre evoked dorsal horn cell activity has been tested at P21 and adult (P40). The results show a marked shift in the selectivity of descending brainstem control of dorsal horn cells from facilitation of A fibre evoked responses at P21 to inhibition of C fibre responses at P40.

## Methods

### Ethical approval

All experiments were conducted in accordance with the United Kingdom Animals (Scientific Procedures) Act of 1986.

Sprague–Dawley rats of both sexes aged P21 and P40 were used in these studies. Animals were allowed free access to water and food and were housed in 12 h light/dark cycles. P21 and P40 rats were caged according to sex in cages of six littermates. A total of 22 P40 rats and 26 P21 rats were used in this study.

Rats were anaesthetized with isoflurane (induction at 3.5% in medical O_2_), tracheotomized and artificially ventilated under constant isoflurane anaesthesia (maintenance of 1.8% in medical O_2_, Univentor Anaesthesia Unit 400; Royem Scientific, Bedfordshire, UK). The air flow was adjusted according to the animal's size using a ventilator pump (small animal ventilator, model 687; Harvard Apparatus Inc, Holliston, MA, USA), and heart rate monitored via electrocardiogram. A homoeothermic blanket and heating lamp were used to maintain body temperature at physiological levels. The rat was mounted on to a stereotaxic frame (Kopf Instruments, Tujunga, CA, USA). A laminectomy was performed to expose the lumbar spinal cord, the vertebral column secured with a clamp to the thoracic spine and the dura and pia mater removed. A subcutaneous injection of saline was given to each animal post laminectomy to maintain hydration and a thin film of mineral oil was used to cover the exposed spinal cord to prevent excessive drying of the cord and heat loss. The skull was exposed and bregma located to perform a small craniotomy for RVM stimulation.

### Compound action potential recordings

Peripheral nerve compound action potential recordings were recorded in P21 and P40 rats to establish the afferent groups excited by peripheral electrical stimulation of the receptive field at the various ages. Rats were anaesthetized, tracheotomized and artificially ventilated as outlined above. The hindlimb was suspended at the ankle and sciatic nerve exposed and isolated by means of a thin film of plastic at the level of the thigh. The nerve was gently freed from the underlying tissue with finely pulled glass hooks and mounted on to silver wire for recording. Filters were set to 0–150 Hz. Two insect pin electrodes were inserted into the ankle 5 mm apart, and current pulses delivered via a stimulus isolator box (NL800, NeuroLog; Digitimer, Welwyn Garden City, Herts, UK) at 1–10 mA amplitude and 50 µs or 500 µs pulse width, to stimulate A and C fibres.

### Rostroventral medulla stimulation

Stereotaxic coordinates for the RVM were calculated as outlined previously (Hathway *et al*. 2009) and a current was applied through a concentric bipolar stimulating electrode, lowered into the RVM. Trains of stimuli of 500 µs pulse width were applied at 10 Hz, at 10 and 100 µA using a stimulus isolator (Neurolog). These stimulus parameters have been shown to evoke reliable descending inhibition and excitation (Zhuo & Gebhart, 1997; Hathway *et al*. 2009). While electrical stimulation may activate fibres of passage as well as neurons in the RVM, this method reliably recruits comparable descending inhibition to microinjections of excitatory amino acids (Zhuo & Gebhart, 1990). Importantly, electrical stimulation allows rapid and reversible investigation of the effects of low-to high-intensity RVM stimulation upon the same dorsal horn cell, and is particularly useful in developmental studies, where populations of cells could vary in sensitivity to excitatory ligands over the course of the postnatal period.

### *In vivo* extracellular recordings in the dorsal horn

To isolate individual neurons in the dorsal horn, a 10 µm tipped glass-coated tungsten microelectrode (Ainsworks, London, UK) was lowered through the cord in 2 or 10 µm steps with a microdrive. Stroking of the plantar skin of the hind paw was used as a search stimulus and when a cell had been isolated, two insect pin electrodes were inserted, 2 mm apart, into the receptive field.

Wide dynamic range cells were selected in the deep dorsal horn, which responded to electrical stimulation to both A and C fibre inputs. For each cell, the thresholds for A fibre and C fibre evoked activity was established using electrical stimulation of the receptive field in pulses of increasing amplitude at 50 µs pulse width (A fibre targeting) and 500 µs pulse width (C fibre targeting) at a frequency of 1 Hz. The A or C fibre electrical threshold was established as the lowest amplitude needed to reliably evoke spikes within 0–50 ms and 50–130 ms latencies respectively. Owing to the difficulty in separating Aβ repetitive firing at short latency (0–5 ms) and Aδ mediated firing at medium latency (5–50 ms), Aβ and Aδ fibre mediated action potential firing and thresholds were grouped and counted together as ‘A fibre mediated’. Trains of 16 A fibre and C fibre strength pulses were applied at 2× threshold and 1 Hz to the receptive field to assess wind-up (Mendell, 1966). Cells were determined to have wound-up if they progressively increased the number of action potentials fired to the repetitive stimulus. Wind-up was quantified by the number of action potentials fired to the 16th C fibre stimulus minus the number of action potentials fired to the first stimulus. Neurons that displayed wind-up would therefore incur a positive wind-up score, whereas neurons that did not would have either a zero or a negative score. Cells that did not display reliable action potential firing to both A-and C-targeted stimulation were excluded from analysis. ROUT analysis was used to exclude outliers with exceptionally high or low thresholds. Nine P40 and five P21 cells were excluded from the final analysis based on these criteria.

### Experimental protocol

Spontaneous activity was recorded for 1 min and then baseline A and C fibre thresholds and ‘wind-up’ were established as described above. Each set of peripheral stimulation was separated by 1 min rest periods to allow for afterdischarges. Next, RVM stimulation (10 µA) was initiated and spontaneous activity, A and C fibre thresholds and ‘wind up’ re-measured. The procedure was repeated during 100 µA RVM stimulation following a 1 min rest period. At the end of the experiment, the same parameters were measured again at baseline, in the absence of any RVM stimulation, to ensure that the experimental protocol had not caused lasting changes to dorsal horn excitability.

Recording and analysis were performed using Chart 5 software (Chart 5 version 5.5.5; ADInstruments Ltd, Oxford, UK) and statistical analyses and graphing were performed using GraphPad Prism (GraphPad Software, version 5.00, La Jolla California USA), Wilcoxon's paired Student's *t* tests or Friedman's paired one-way ANOVAs were used within an age group followed by Dunnett's *post hoc* multiple comparisons test for significant values. For all data, a 95% confidence interval was used as a measure of statistical significance. Data are presented as means ± s.e.m.

All animals were killed with an overdose of isoflurane at the end of the experiment.

## Results

A total of 46 wide dynamic range deep dorsal horn cells with both A fibre and C fibre evoked activity were recorded, 26 of these from P21 rat pups and 20 from P40 rats. Neurons in laminae IV–V were used for this study as they receive a high density of descending input from the brainstem (Todd, 2010); the mean recording depth at P21 was 524 ± 28 µm (*n* = 26) and 481 ± 35 µm (*n* = 20) at P40. All neurons responded to both low-intensity brush and high-intensity pinch of the hindpaw skin and responded with a clearly separable short latency A fibre, and longer latency C fibre evoked activity (Fig. 1). Latencies of Aβ, Aδ and C fibre compound action potentials were recorded from the dorsal roots at both ages to confirm electrical thresholds and conduction velocities at the two ages. Wind-up to repeated electrical stimulation of the receptive field at C fibre intensity was observed in 17 (59%) of the recorded cells at P21 and in eight (40%) of the cells at P40. No wind-up was observed upon repeated receptive field stimulation at A fibre intensity.

**Figure 1:**
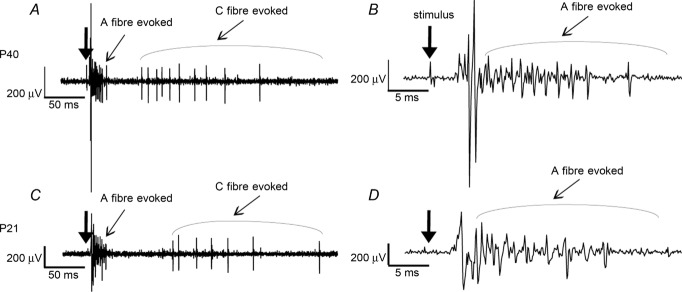
*A*, typical response from a P40 rat WDR neuron: a short latency burst of action potentials evoked by A fibre afferents is followed by a longer latency train of action potentials evoked by C fibre afferents. *B*, same trace as (*A*) but on a longer time scale to show A fibre evoked train. *C*, typical response from a P21 rat WDR neuron: the same short latency A fibre evoked and long latency C fibre evoked responses are observed as at P40. *D*, same trace as (*C*) but on a longer time scale to show the A fibre evoked train. The electrical pulse was delivered to the receptive field at the time marked by the vertical arrow. P21, postnatal day 21; P40, postnatal day 40; WDR, wide dynamic range.

### Inhibition of C fibre evoked activity from the rostroventral medial medulla in adult but not young rats

The effect of RVM activation upon the electrical threshold for evoking A fibre and C fibre responses in dorsal horn cells was established at P21 and P40. The threshold was classed as facilitated or inhibited by RVM stimulation if it decreased or increased by >10% of baseline values.

The threshold for C fibre evoked activity in P40 rats was increased by a mean of 50% from baseline during 100 µA RVM stimulation, while 10 µA stimulation did not significantly affect this measure [baseline (BL), 3.0 ± 0.3 mA; 10 µA, 3.5 ± 0.6 mA; 100 µA, 4.9 ± 0.8 mA; repeat baseline, 4.3 ± 0.7 mA; *n* = 19; one-way ANOVA Friedman test with Dunn's multiple comparisons: *P* = 0.0001, BL *vs*. 100 µA: mean increase 49.62 ± 17.32%; *P* < 0.0001; Fig. 2*C* and *D*].

**Figure 2:**
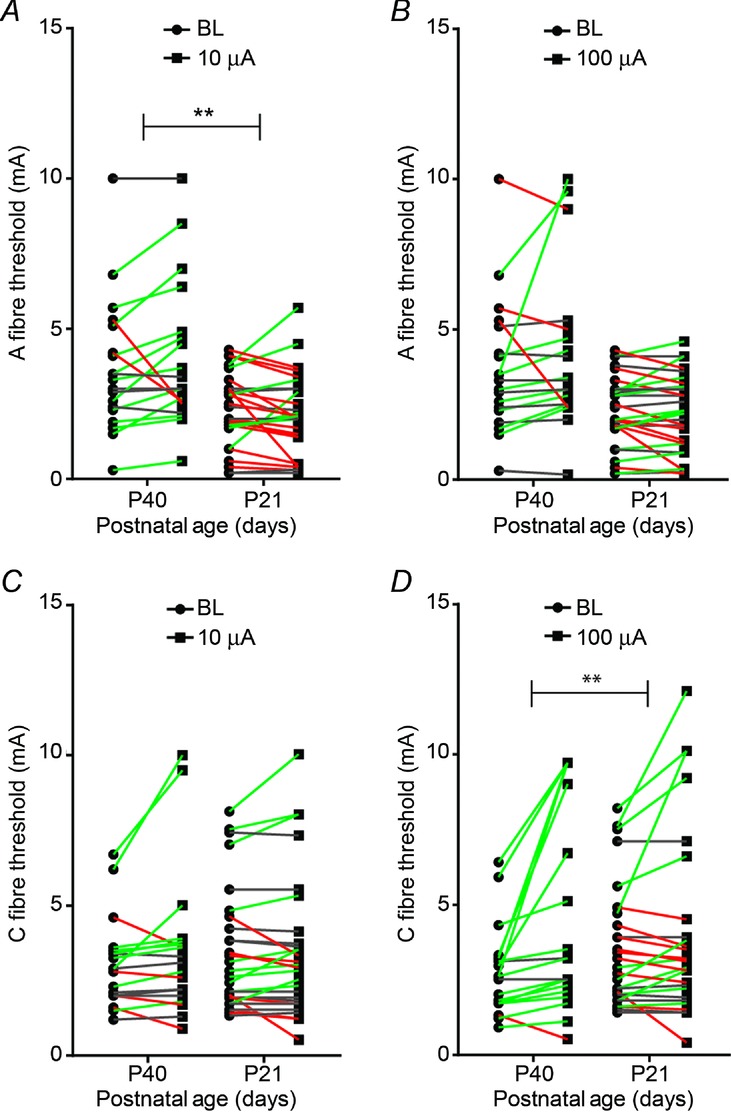
*A* and *B*, effect of 10 µA and 100 µA RVM stimulation on the thresholds for A fibre evoked activity, while (*C*) and (*D*) show the effects of 10 µA and 100 µA RVM stimulation on the thresholds for C fibre evoked activity. For illustrative purposes, cells in which RVM stimulation caused an increase in threshold from BL (>10%) are shown in green (inhibition), cells in which there was a decrease in threshold (>10%) in red (excitation) and cells with no change (≤10%) in grey. *A*, 10 µA RVM stimulation predominantly increased the threshold (green) for A fibre evoked activity at P40, but decreased it (red) at P21 (χ^2^ analysis comparing P40 and P21 neuronal responses at 10 µA RVM stimulation and A fibre peripheral stimulation, *P* = 0.002). *B*, effects of 100 µA RVM stimulation on the threshold for A fibre evoked activity were mixed and not significantly different at P21 and P40. *C*, effects of 10 µA RVM stimulation upon the threshold for C fibre evoked activity were mixed and not significantly different at P21 and P40. *D*, 100 µA RVM stimulation predominantly increased the threshold (green) for C fibre evoked activity at P40, but not at P21 (χ^2^ analysis comparing P40 and P21 neuronal responses at 100 µA RVM stimulation and C fibre peripheral stimulation, *P* = 0.001). P21, *n* = 26; P40, *n* = 20. BL, baseline; P21, postnatal day 21; P40, postnatal day 40; RVM, rostroventral medial medulla.

In contrast, RVM stimulation at P21 had no significant effect upon C fibre evoked activity threshold at either 10 or 100 µA stimulation intensities (BL, 3.3 ± 0.4 mA; 10 µA, 3.4 ± 0.4 mA; 100 µA, 3.8 ± 0.2 mA; repeat BL, 3.8 ± 0.5 mA; χ^2^ analysis comparing P40 and P21 neuronal responses at 100 µA RVM stimulation and C fibre peripheral stimulation, *P* = 0.001; Fig. 2*C* and *D*). Importantly the number of C fibre evoked spikes at threshold was not significantly different between the two ages at baseline (P21 baseline, 9.1 ± 1.2 spikes s^–1^ *n* = 29; P40 baseline, 5.3 ± 0.8 spikes s^–1^ *n* = 19; Mann–Whitney test, *P* = 0.06; data not shown). Thus, high-intensity RVM stimulation decreases the likelihood of C fibre inputs evoking activity in the dorsal horn of adult rats but not young rats.

### Facilitation of A fibre evoked activity from the rostroventral medial medulla in young but not adult rats

While RVM stimulation showed little effect upon C fibre thresholds in the adolescent dorsal horn, 10 µA RVM stimulation at P21 significantly decreased the threshold for A fibre evoked activity, without any detectable effects on threshold arising following 100 µA stimulation (BL, 2.3 ± 0. 2 mA; 10 µA, 2.1 ± 0.2 mA; 100 µA, 2.2 ± 0.2 mA; repeat BL, 2.4 ± 0.3 mA; one-way ANOVA Friedman test, *P* = 0.05; Fig. 2*A*). This was in contrast to the effect on adult rats (P40), where RVM stimulation significantly increased A fibre threshold at 10 µA intensity, without any detectable effect occurring during 100 µA stimulation (BL, 3.7 ± 0.5 mA; 10 µA, 4.0 ± 0.6 mA; 100 µA, 4.18 ± 0.6 mA; repeat BL, 3.9 ± 0.5 mA; Dunn's multiple comparison test: BL *versus* 10 µA, *P* = 0.03; Fig. 2*A* and *B*; χ^2^ analysis comparing P40 and P21 neuronal responses at 10 µA RVM stimulation and A fibre peripheral stimulation, *P* = 0.002). Thus, low-intensity RVM stimulation increases the likelihood of A fibre evoked activity in the dorsal horn of young rats while decreasing this likelihood in the adult dorsal horn.

### Rostroventral medial medulla descending control of individual dorsal horn neurons depends upon their state of excitability

The state of excitability of a dorsal horn neuron can be measured, at least in part, by whether it winds up to repeated C fibre stimulation. To test whether the effect of RVM stimulation was related to the excitability of individual neurons, we plotted the change in A and C fibre threshold during RVM stimulation against the degree of wind up at baseline. At P40, wind-up was significantly correlated to a change in C fibre threshold during 10 µA, but not 100 µA, RVM stimulation (*r*^2^ = 0.23; *P* = 0.04; Fig. 3*B*). No such correlation was found in P21 neurons (Fig. 3*D*). Thus, in the adult, although the mean C fibre threshold of the whole population of dorsal horn neurons was not significantly altered by low-intensity 10 µA RVM stimulation, descending modulation depends upon the state of excitability of individual dorsal horn neurons; the C fibre input to more excitable neurons is inhibited while the C fibre input to less excitable ones is facilitated. This relationship was not observed at P21. Importantly, C fibre wind-up firing in itself was not significantly affected by RVM stimulation at either age (P40 mean area under the curve BL, 111.2 ± 41.8; 10 µA, 96.6 ± 18.3; 100 µA, 76.6 ± 40.8; repeat BL, 99.1 ± 41.7; one-way ANOVA *P* = 0.16; P21 mean BL, 197.9 ± 19.5; 10 µA, 192.8 ± 23.3; 100 µA, 191.7 ± 24.3; repeat BL, 186.8 ± 28.8; *P* = 0.73; Fig. 4).

**Figure 3:**
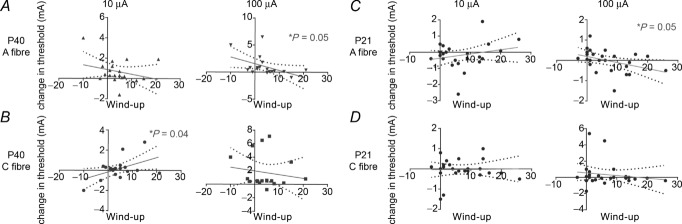
For each cell the change in threshold for A fibre (*A* and *C*) and C fibre (*B* and *D*) evoked activity during 10 µA (left panel) and 100 µA (right panel) RVM stimulation is plotted against the wind-up at P40 (*A* and *B*) and P21 (*C* and *D*). Wind-up is measured as the number of action potentials evoked by the 16th C fibre stimulus minus the number evoked by the first stimulus at baseline. *A*, at P40, a fall in threshold for A fibre evoked activity caused by 100 µA, but not 10 µA, RVM stimulation is correlated with wind-up. *B*, at P40, the rise in threshold for C fibre evoked activity caused by 10 µA, but not 100 µA, RVM stimulation is correlated with greater wind-up. *C*, at P21, the fall in threshold for A fibre evoked activity caused by 100 µA, but not 10 µA, RVM stimulation is correlated with greater wind-up. *D*, at P21, the change in threshold for C fibre evoked activity is not correlated to wind-up. P21, *n* = 26; P40, *n* = 20. Correlations: (*A*) 100 µA, *r*^2^ = 0.21, slope = −0.12, *P* = 0.05; (*B*) 10 µA, *r*^2^ = 0.23, slope = 0.07, *P* = 0.04; (*C*) 100 µA, *r*^2^ = 0.16, slope = −0.03, *P* = 0.05. P21, postnatal day 21; P40, postnatal day 40; RVM, rostroventral medial medulla.

**Figure 4:**
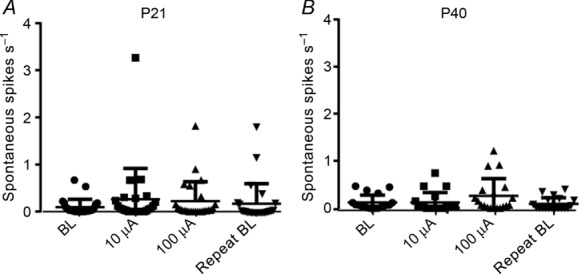
Activity is plotted at BL, during 10 µA and 100 µA rostroventral medial medulla stimulation and at recovery (repeat BL). Rostroventral medial medulla stimulation had no significant effect upon spontaneous firing at (*A*) P21 (*n* = 26) or at (*B*) P40 (*n* = 20). P21: one-way ANOVA Friedman test, *P* = 0.31; P40, *P* = 0.47. BL, baseline; P21, postnatal day 21; P40, postnatal day 40.

At P21, wind-up was significantly correlated to a change in A threshold during 100 µA, but not 10 µA, RVM stimulation (*r*^2^ = 0.16, *P* = 0.05; Fig. 3*C*). This correlation was also present at P40 (*r*^2^ = 0.21, *P* = 0.05; Fig. 3*A*). Thus at both P21 and P40, change in A fibre threshold during high-intensity RVM stimulation is reliant upon the activity level of the cell. Therefore, although the mean A fibre threshold of the whole population of dorsal horn neurons was not significantly altered by high-intensity 100 µA RVM stimulation at either P21 or P40, descending modulation depends upon the state of excitability of individual dorsal horn neurons; the A fibre input to more excitable neurons is facilitated while the A fibre input to less excitable ones is inhibited.

Spontaneous firing was unaffected by RVM stimulation at either P21 [BL, 0.09 ± 0.03 spikes s^–1^; 10 µA, 0.25 ± 0.13 spikes s^–1^; 100 µA, 0.2 ± 0.08 spikes s^–1^; repeat BL, 0.2 ± 0.09 spikes s^–1^ (one-way ANOVA *P* = 0.31)] or P40 [BL, 0.12 ± 0.04 spikes s^–1^; 10 µA, 0.12 ± 0.05 spikes s^–1^; 100 µA, 0.3 ± 0.08 spikes s^–1^; repeat BL, 0.1 ± 0.03 spikes s^–1^ (one-way ANOVA *P* = 0.47)] (Fig. 5*A* and *B*), nor was susceptibility to descending modulation related to the spontaneous firing of the recorded neurons (data not shown). Descending modulatory influence from the RVM is therefore not dependent upon baseline activity in the dorsal horn circuitry.

**Figure 5:**
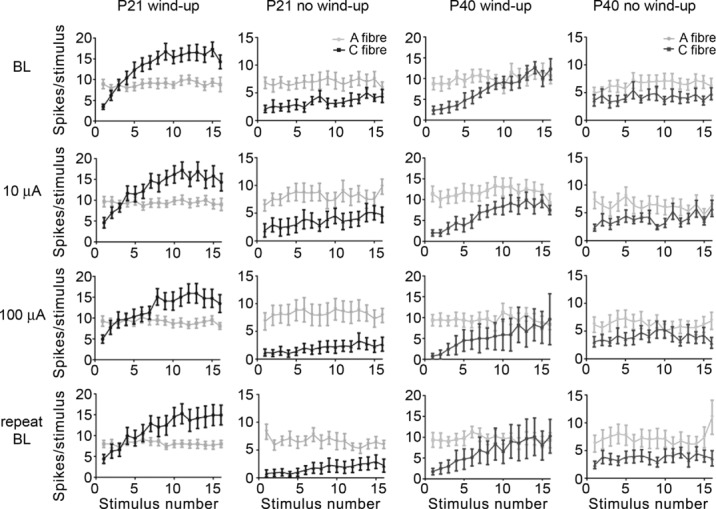
Each point represents the mean number of C fibre evoked spikes (±s.e.) fired to each of 16 electrical pulses applied to the skin of the receptive field. For illustrative purposes cells are divided into those that displayed wind-up (P21, *n* = 17; P40, *n* = 8) and those that did not (P21, *n* = 9; P40, *n* = 12) at the two ages. The top panel shows wind-up at BL, the next panels during 10 µA and then 100 µA rostroventral medial medulla stimulation, and the bottom panel at recovery (repeat BL). Rostroventral medial medulla stimulation at 10 µA or 100 µA does not affect the wind-up of dorsal horn wide dynamic range neurons to repeated A fibre or C fibre peripheral stimulation. Area under the curve analysis: P40, C fibre wind-up, one-way ANOVA Friedman test, *P* = 0.16; A fibre, *P* = 0.24; P21, C fibre wind-up, one-way ANOVA Friedman test, *P* = 0.73; A fibre, *P* = 0.13. BL, baseline; P21, postnatal day 21; P40, postnatal day 40.

## Discussion

Here we describe how the balance of inhibition and facilitation of RVM descending control over dorsal horn neurons is dependent on afferent evoked activity, the excitability state of the dorsal horn neuron and the age of the animal. It is clear that there is no ‘absolute’ state of descending control over spinal nociceptive circuits, but rather a control that is tailored to particular dorsal horn activity at a particular developmental stage. We have shown that in the mature, P40, spinal dorsal horn, descending inhibition from the RVM is targeted to inhibit C fibre input, in contrast to the adolescent, P21, spinal cord, where instead there is a descending facilitation of A fibre inputs. However, in individual neurons, facilitation and inhibition is, at least in part, dependent upon their excitability, as measured by their degree of C fibre wind-up at baseline.

Change in threshold is often used in pain research as a measure of change in sensitivity to a noxious stimulus. The experiments described here examine changes in the A and C fibre electrically evoked threshold following RVM stimulation because it allowed precise measurement and comparison across ages and RVM stimulus strengths and can be validated from compound action potential recordings. In agreement with previous reports (Lu *et al*. 2004) we found that 100 µA stimulation of the RVM in adults rats increased the threshold and therefore inhibited C fibre evoked activity in the dorsal horn population as a whole. Previous reports have shown that adult RVM stimulation at 10 µA is facilitatory (Zhuo & Gebhart, 1997), and while we did not observe this in the mean population, we did find that neurons that do not display C wind-up are more likely to have decreased C thresholds (i.e. be facilitated) by 10 µA RVM stimulation, suggesting that the facilitatory effects described in previous studies could have been in a less excited set of cells. Our results suggest that whereas high-intensity stimulation of the RVM is strong enough to inhibit C fibre input in most neurons, low-intensity 10 µA stimulation is more plastic, and can facilitate or inhibit C fibre threshold dependent on the state of the neuron.

Interestingly, while we found a positive correlation between C threshold change and wind-up at 10 µA RVM stimulation, there was a negative correlation between A threshold change and wind-up at high-intensity 100 µA RVM stimulation, whereby neurons displaying wind-up at baseline had a decreased A fibre threshold. This could be particularly relevant given the presumed role of both descending control and spinal wind-up in pain states (Herrero *et al*. 2000; Sandkühler, 2009), and could be a novel mechanism for plastic changes underlying the hyperalgesia and allodynia associated with pathological pain. Thus cells with increased wind-up following tissue inflammation (Stanfa *et al*. 1992; Traub, 1997) or induction of monoarthritic pain (Herrero & Cervero, 1996), may trigger increased RVM activity, which in turn results in descending facilitation of their A fibre input. While increased induction of this spinobulbospinal loop is a possible mechanism in the intact awake animal, this mechanism has not been tested herein, where both descending and peripheral inputs were stimulated.

In agreement with previous studies, we found that the overall effect of stimulation of the RVM in P21 adolescent rats upon dorsal horn cells was facilitatory (Hathway *et al*. 2009). However, we show here that this facilitation is targeted to the A fibre input only, decreasing A fibre threshold but not affecting the C fibre input threshold of these cells. At 100 µA RVM stimulation descending facilitation of the A threshold on P21 neurons was correlated with the excitability of the neuron, as measured by C fibre wind-up. Although the magnitude of RVM A fibre facilitation of adolescent dorsal horn neurons was small, it was consistent and reproducible across individual recordings indicative of a functionally relevant result. Interestingly, a drop in electrical pain threshold of 200 µA, as we describe here, can readily be detected by human subjects and is reflected in both heat and mechanical pain scores, suggesting this decrease in threshold is likely to have behavioural significance in the awake behaving animal (Weinkauf *et al*. 2013).

A recent study by Hellman and Mason (2012) presented the RVM as a salience detector, acting to modulate nociceptive stimuli specifically within the dorsal horn, and not, as previously thought, to control basal ongoing activity in spinal circuits. In agreement with this, we found that the strength of descending modulation was not correlated to the inherent spontaneous, or basal, activity of the neuron at either age tested. Instead, brainstem modulatory control was correlated to the degree of C fibre wind-up, activity that is dependent upon nociceptive-specific afferent input. We propose, following this model, that descending modulation of dorsal horn circuits is tuned to modulate afferent input to which the animal is normally salient within a nociceptive context. In accordance with this, nociceptive activity in the mature dorsal horn is predominantly C fibre stimulus-driven and functional magnetic resonance imaging studies in humans have shown evidence for C fibre selective salience within the insula (Weiss *et al*. 2008). In contrast, activity in the immature dorsal horn is predominantly A fibre driven (Park *et al*. 1999; Nakatsuka *et al*. 2000), and we therefore hypothesize that salience is focused to A fibre nociceptive activity in young animals. Facilitation of A fibre inputs in the immature cord by the RVM may serve to increase dorsal horn activity and reinforce synapses, particularly those from newly formed nociceptive inputs, enhancing the maturation of nociceptive networks and reflexes over this critical period.

## Key points

- Brainstem descending pathways control the balance of excitation and inhibition in spinal sensory networks. In adult rodents, descending inhibition is targeted to spinal neurons with a strong afferent C fibre input.
- Descending inhibitory control matures slowly; the first postnatal weeks are characterized by greater descending facilitation than inhibition.
- We report that, in contrast to adults, brainstem descending facilitation of spinal sensory neurons in young rats (postnatal day 21) is targeted to A fibre inputs. The selective inhibition of C fibre inputs observed in adults is absent at postnatal day 21.
- In both young and adult rats, descending inhibition or facilitation is correlated with the excitability of individual neurons, as measured by ‘wind-up’ to repeated C fibre stimulation.
- The facilitation of A fibre input in early life is likely to enhance innocuous, tactile sensory inputs to the dorsal horn in the critical early postnatal weeks and thus promote activity-dependent development of sensory networks.

## Abbreviations

BL: baseline
P21, P40: postnatal day 21, postnatal day 40
RVM: rostroventral medial medulla

## References

[b1] Aicher SA, Hermes SM, Whittier KL, Hegarty DM (2012). Descending projections from the rostral ventromedial medulla (RVM) to trigeminal and spinal dorsal horns are morphologically and neurochemically distinct. J Chem Neuroanat.

[b2] Basbaum AI, Fields HL (1984). Endogenous pain control systems: brainstem spinal pathways and endorphin circuitry. Annu Rev Neurosci.

[b3] Beggs S, Torsney C, Drew LJ, Fitzgerald M (2002). The postnatal reorganization of primary afferent input and dorsal horn cell receptive fields in the rat spinal cord is an activity-dependent process. Eur J Neurosci.

[b4] Cervero F, Plenderleith MB (1985). C-fibre excitation and tonic descending inhibition of dorsal horn neurones in adult rats treated at birth with capsaicin. J Physiol.

[b5] Fields HL, Basbaum AI, Clanton CH, Anderson SD (1977). Nucleus raphe magnus inhibition of spinal cord dorsal horn neurons. Brain Res.

[b6] Fields HL, Basbaum AI, Heinricher MM, McMahon S, Koltzenburg M (2006). Central nervous system mechanisms of pain modulation. Wall and Melzack's Textbook of Pain.

[b7] Fitzgerald M, Koltzenburg M (1986). The functional development of descending inhibitory pathways in the dorsolateral funiculus of the newborn rat spinal cord. Brain Res.

[b8] Gebhart GF, Sandkuhler J, Thalhammer JG, Zimmermann M (1983). Quantitative comparison of inhibition in spinal cord of nociceptive information by stimulation in periaqueductal grey or nucleus raphe magnus of the cat. J Neurophysiol.

[b9] Granmo M, Petersson P, Schouenborg J (2008). Action-based body maps in the spinal cord emerge from a transitory floating organization. J Neurosci.

[b10] Hathway GJ, Koch S, Low L, Fitzgerald M (2009). The changing balance of brainstem-spinal cord modulation of pain processing over the first weeks of rat postnatal life. J Physiol.

[b11] Hellman KM, Mason P (2012). Opioids disrupt pro-nociceptive modulation mediated by raphe magnus. J Neurosci.

[b12] Herrero JF, Cervero F (1996). Changes in nociceptive reflex facilitation during carrageenan-induced arthritis. Brain Res.

[b13] Herrero JF, Laird JMA, Lopez-Garcia JA (2000). Wind-up of spinal cord neurones and pain sensation: much ado about something?. Prog Neurobiol.

[b14] Kwok CH, Devonshire IM, Bennett AJ, Hathway GJ (2013). Postnatal maturation of endogenous opioid systems within the periaqueductal grey and spinal dorsal horn of the rat. Pain.

[b15] Lu Y, Sweitzer SM, Laurito CE, Yeomans DC (2004). Differential opioid inhibition of C-and Aδ-fiber mediated thermonociception after stimulation of the nucleus raphe magnus. Anesth Analg.

[b16] McMullan S, Lumb BM (2006). Spinal dorsal horn neuronal responses to myelinated *versus* unmyelinated heat nociceptors and their modulation by activation of the periaqueductal grey in the rat. J Physiol.

[b17] Mendell LM (1966). Physiological properties of unmyelinated fibre projection to the spinal cord. Exp Neurol.

[b18] Nakatsuka T, Ataka T, Kumamoto E, Tamaki T, Yoshimura M (2000). Alteration in synaptic inputs through C-afferent fibers to substantia gelatinosa neurons of the rat spinal dorsal horn during postnatal development. Neuroscience.

[b19] Odeh F, Antal M (2001). The projections of the midbrain periaqueductal grey to the pons and medulla oblongata in rats. Eur J Neurosci.

[b20] Park JS, Nakatsuka T, Nagata K, Higashi H, Yoshimura M (1999). Reorganization of the primary afferent termination in the rat spinal dorsal horn during post-natal development. Brain Res Dev Brain Res.

[b21] Porreca F, Ossipov MH, Gebhart GF (2002). Chronic pain and medullary descending facilitation. Trends Neurosci.

[b22] Prieto GJ, Cannon JT, Liebeskind JC (1983). N. raphe magnus lesions disrupt stimulation-produced analgesia from ventral but not dorsal midbrain areas in the rat. Brain Res.

[b23] Sandkühler J (2009). Models and mechanisms of hyperalgesia and allodynia. Physiol Rev.

[b24] Sandkuhler J, Gebhart GF (1984). Characterization of inhibition of a spinal nociceptive reflex by stimulation medially and laterally in the midbrain and medulla in the pentobarbital-anaesthetized rat. Brain Res.

[b25] Stanfa LC, Sullivan AF, Dickenson AH (1992). Alterations in neuronal excitability and the potency of spinal mu, delta and kappa opioids after carrageenan-induced inflammation. Pain.

[b26] Tavares I, Lima D (1994). Descending projections from the caudal medulla oblongata to the superficial or deep dorsal horn of the rat spinal cord. Exp Brain Res.

[b27] Todd AJ (2010). Neuronal circuitry for pain processing in the dorsal horn. Nat Rev Neurosci.

[b28] Todd AJ, McGill MM, Shehab SA (2000). Neurokinin 1 receptor expression by neurons in laminae I, III and IV of the rat spinal dorsal horn that project to the brainstem. Eur J Neurosci.

[b29] Traub RJ (1997). Spinal modulation of the induction of central sensitization. Brain Res.

[b30] Waters AJ, Lumb BM (2008). Descending control of spinal nociception from the periaqueductal grey distinguishes between neurons with and without C-fibre inputs. Pain.

[b31] Weinkauf B, Main M, Schmelz M, Rukwied R (2013). Modality-specific nociceptor sensitization following UV-B irradiation of human skin. J Pain.

[b32] Weiss T, Straube T, Boettcher J, Hecht H, Spohn D, Miltner WHR (2008). Brain activation upon selective stimulation of cutaneous C-and Aδ-fibers. NeuroImage.

[b33] Zhuo M, Gebhart GF (1990). Characterization of descending inhibition and facilitation from the nuclei reticularis gigantocellularis and gigantocellularis pars alpha in the rat. Pain.

[b34] Zhuo M, Gebhart GF (1994). Effects of neonatal capsaicin treatment on descending modulation of spinal nociception from the rostral, medial medulla in adult rat. Brain Res.

[b35] Zhuo M, Gebhart GF (1997). Biphasic modulation of spinal nociceptive transmission from the medullary raphe nuclei in the rat. J Neurophysiol.

